# Mycological Pattern of Dermatomycoses in a Tertiary Care Hospital

**DOI:** 10.1155/2015/157828

**Published:** 2015-09-30

**Authors:** Ravinder Kaur, Pragyan Swagatika Panda, Kabir Sardana, Sahanawaj Khan

**Affiliations:** ^1^Department of Microbiology, Lady Hardinge Medical College, New Delhi 110001, India; ^2^Department of Microbiology, Maulana Azad Medical College & Associated Hospitals, New Delhi 110002, India; ^3^Department of Dermatology & Sexually Transmitted Diseases, Maulana Azad Medical College & Associated Hospitals, New Delhi 110002, India

## Abstract

*Background*. Dermatomycoses are not diseases requiring compulsory notifications; rather they cause cosmetic defacements. Indian subcontinent with a varied topography is favorable for various fungal infections. *Objective*. To look for the epidemiological and mycological profile of superficial mycoses in North India. *Methods*. Three hundred and fifty-one clinical samples of skin, hair, and nail were examined to find the fungal etiology of the dermatomycoses. *Results*. Dermatomycoses were seen in 215/351 (61.2%) of cases. Most common isolates obtained were nondermatophyte molds (NDMs) (36.1%), followed by dermatophytes (13.8%) and yeasts (8.6%). *Aspergillus niger* (9%) was the most common mold. *Trichophyton rubrum* (4.6%) was the most common dermatophyte isolated, while amongst the yeasts *Non-albicans Candida* (NAC) species were more common (6%). Many other NDMs like *Syncephalastrum* spp., *Cunninghamella* spp., *Rhodotorula* spp., *A. terreus, Scytalidium* spp. and *Scopulariopsis* spp. were also isolated. *Conclusion*. Our study reflects an increasing role of NDMs (thought to be normal laboratory or environmental contaminants) as a causative agent of dermatomycoses, replacing the dermatophytes. Clinician's awareness of the demographic profile of the population involved along with more studies on dermatomycoses can help in understanding the etiological profile in area, leading to prevention of disease occurrence and cosmetic disfigurement.

## 1. Introduction

Fungal infections, very common in man, are of an increasing significance both in developed and developing countries due to illnesses like diabetes, HIV infection, and the use of immunosuppressive drugs. In dermatology outpatient clinics, principally in tropical countries, cases of tinea (cutaneous/skin mycoses) and other superficial fungal infections observed daily with definitive diagnoses can be detected on the skin, in hair, on the nails, in periungual folds, in the mucosa, and so forth [1]. It is difficult to outline their exact epidemiological profile as these are not diseases requiring compulsory notifications [2]. Several factors affect the higher incidence of superficial and cutaneous mycoses, including bioclimatic conditions favorable to the development of fungi in saprophytic life, promiscuity, sweating, prolonged contact with pets, and contaminated water from swimming pools and surrounding risk areas [1]. Superficial mycoses are infections of skin, hairs, and nails caused by dermatophytes, yeasts, and nondermatophyte molds. Amongst these, dermatophytes are responsible for the largest number of cases [3]. The Indian subcontinent has a varied topography and the hot and humid climate is highly favorable for the acquisition of fungal infections. The nature of dermatomycoses changes with the passage of time, change in living conditions, and evolution of preventive measures and hygienic conditions in society [4]. These variations inspired us to study the epidemiological and mycological pattern of dermatomycoses to contribute to the current understanding of the etiological distribution of the most frequent fungal agents causing the skin, hair, and nail infections in our region.

## 2. Materials and Methods

The study was conducted in the Department of Microbiology, Maulana Azad Medical College, New Delhi, over a period of two years (2012-2013). The study group comprised three hundred and fifty-one (351) clinically suspected cases of dermatomycoses, and the hair, skin, and nail samples were collected as per individual's symptomatology. The samples were collected in sterile petri dishes and direct microscopy using 10%–20% potassium hydroxide (KOH) was done and the samples were screened under 10x and 40x for fungal hyphae, spores, or yeast cells. They were then inoculated onto two sets of Sabouraud dextrose agar (SDA) containing chloramphenicol (0.05 mg/mL) and cycloheximide (0.1–0.4 mg/mL) and incubated at 25°C and 37°C. The cultures were examined once a week and were declared negative if no growth was obtained till 4–6 weeks.

The isolates were further identified by studying the culture characteristics, pigment production, and microscopic examination of the lactophenol cotton blue (LPCB) mounts and slide culture (wherever necessary). Those samples that yielded 3 or more growth and were negative in KOH mount were considered contaminants/mixed growth. Two different fungal structures seen on microscopy and corresponding two different fungi isolated on first and repeat cultures were termed as double growth. Mixed growth was defined as isolation of more than or equal to 3 fungal growth on SDA, without positive findings in the KOH mount. Contaminants were defined as fungal growth on SDA without a positive KOH mount or no growth on repeat culture or negative on repeat KOH mount or mixed growth.

## 3. Result

Of these 351 cases, 236 (67.2%) were males and 115 (32.7%) were females (Table 1) with a male : female ratio of 2 : 1. The commonest age group was 21–30 years (23.3%) followed by 31–40 years (20.5%). In females, it was more common in children <10 years of age (23.4%). Infections were least common in >60 years of age group (5.6%). Patients from urban area (81.5%) were more frequent than those from the rural area (18.5%) and the frequency of literate patients (64.6%) was more than that of the illiterates (35.3%).

Out of all the samples received, 196 (55.8%) were nail samples, 123 (35%) were skin samples, and 32 (9.1%) were hair samples. Amongst the skin samples, 48 (39%) were positive by culture and 44 (35.7%) were positive by KOH. Only 32 (26%) were positive by both KOH and culture. In hair samples, 9 (28.1%) were positive by culture and 8 (25%) were positive by KOH, while 6 (18.7%) were positive by both KOH and culture. In nail samples, 144 (73.4%) were positive by culture and 136 (69.3%) were positive by KOH and 109 (55.6%) were positive by both KOH and culture (Table 2).

Dermatomycoses were seen in 215/351 (61.2%) of cases and 15 (4.2%) of them had double growth, while 9 (2.5%) had mixed growth. Out of 196 nail samples, 144 (73.4%) were single type of growth and 11 (5.6%) were double type of growth, while 5 (2.5%) were contaminants. Out of 123 skin samples, 48 (39%) had single growth, 4 (3.2%) had double growth, and 4 (3.2%) had mixed growth. Out of 32 hair samples, 9 (28.1%) had single isolate growth. There was no growth in 54.4% of skin samples, 71.8% of hair samples, and 19.8% of nail samples (Table 3).

Most common isolates obtained in our study were nondermatophyte molds (NDMs) (36.1%), followed by dermatophytes (13.8%) and yeasts (8.6%). Amongst the NDMs,* Aspergillus niger* (9%) was the most common mold, followed by* Aspergillus flavus *(8.1%),* Penicillium *spp. (3.5%),* Mucor *spp. (3.2%),* Rhizopus *spp. (2.7%), and* Alternaria alternata* (2.4%). Other NDMs which were isolated were* Fusarium *spp.,* Cunninghamella *spp.,* Syncephalastrum *spp.,* Bipolaris *spp.,* Scytalidium *spp., and* Curvularia *spp., as shown in Table 4. Amongst the dermatophytes,* Trichophyton rubrum* (4.6%) was the most common followed by* T. verrucosum* (3.2%), while amongst the yeasts* Non-albicans Candida* (NAC) species were more common (6%) as compared to* Candida albicans *(2.1%). From the skin samples,* T. rubrum* (6.2%) was mostly isolated followed by* C. albicans* (5.5%), NAC (5.5%), and* T. schoenleinii* (3.9%). Amongst the hair samples, dermatophytes were mostly isolated (*T. rubrum*,* T. mentagrophytes*, and* T. verrucosum *(6.2% each)). Meanwhile in nail samples most common isolates were* A. niger* (14%),* A. flavus *(12.5%), and NAC (6.7%).* T. rubrum* (3.3%) was the most common dermatophyte isolated followed by* T. verrucosum* (2.8%) (Table 4).

## 4. Discussion

The maximum number of cases was seen in the age group of 21–30 years (23.3%) followed by 31–40 years (20.5%). Similar to our study, a high prevalence in the younger age group was found in another study conducted in Gangtok in 2009 and in Bangalore in 2000 in our country [5, 6]. This increased incidence in the younger population could be because younger patients are more often exposed to occupation-related trauma and increased indoor/outdoor activity. There is also an increased cosmetic consciousness in younger people compared to older people with an increased outpatient visit. Most samples were obtained from male patients (67.2%) as compared to females (32.7%). Similar peak in this age group and a male predominance have been observed by workers in Shimla, India, in 2014 [4]. However in a study in Western Iran in 2013 there was more of female preponderance [7]. The males might be more prone because of prolonged outdoor activity and increased perspiration which creates an environment leading to the development of these fungal infections. In our study 81.5% patients were from urban area, while only 18.5% of patients were from rural area. It might be due to more awareness, literacy, and cosmetic consciousness in urban people as compared to rural people. Most of our patients were literate, which again reflects increased awareness and cosmetic consciousness in them.

Dermatomycoses were seen in 61.5% of cases in our study (Table 2), while in a study in Tamil Nadu in India in 2015 the prevalence of dermatomycoses was 27.6%, which was less as compared to ours [8]. In a study by Veer et al. [9] in Aurangabad, India, dermatomycoses of nail were seen in 48.86% of cases which was also less than the percentage in our study. We found nail as the commonest (55.8%) site of superficial fungal infection followed by skin (35%) and hair (9.1%); Lakshmanan et al. [8] in 2015 had found skin as the commonest site of superficial infection, followed by nail and hair.

Out of 351 samples, 53.5% were positive by KOH and 61.2% were positive by culture. Amongst these, 35.7%, 25%, and 69.3% of skin, hair, and nail samples were positive by KOH, while 39%, 28.1%, and 73.4% of them were positive by culture, respectively. Studies have shown that the KOH wet mount positivity rates range from 23.8% to as high as 91.2% which was true in our case [4]. In a study conducted in Ludhiana 13.2% of KOH negative nail samples exhibited growth on culture which was less as compared to our observations [10]. In our study 53.5% of samples were positive by KOH and 61.2% were positive by culture. In a study in Kashmir, India, in 2013, KOH mount and culture showed positive results in 84 (56%) and 60 (40%) patients, respectively [11]. Shenoy et al. [12] in their study in 2008 showed positive results in 53% and 35% of cases by microscopy and culture, respectively. Meanwhile in the study by Das et al. [13] in eastern part of India in 2008, direct microscopy was positive in only 32.9% of cases, and culture was positive in 49.4% of cases. Our study showed culture was positive in more number of cases as compared to the above-mentioned studies. The skills involved in techniques of sampling and in examining the KOH mount might be different at different places, which might account for the difference in microscopic and culture findings, making it essential that all the KOH negative samples should be cultured.

The predominant isolates in our study were NDMs (36.1%) followed by dermatophytes (13.8%) and yeasts (8.6%). Nondermatophyte molds (NDMs) are considered as pathogenic when they are positive both in microscopy and in culture or if the same isolate is obtained on repeat culture. Collection techniques have been taken care of and proper training for the same has been provided to the concerned people at the collection end and hence a proper collection of sample by the clinician is understood to have been done. Additionally, proper biosafety precautions and care are taken in the laboratory while processing the samples to avoid the growth of laboratory contaminants. In addition there is no increased growth of these NDMs in other samples being processed in the lab to suggest contamination. Studies conducted by Lakshmanan et al. [8] in 2015 and Patel et al. [14] in 2010 in Gujarat (India) also showed an increased prevalence of NDM. However, in a study by Lone et al. [11] in Kashmir, India, the most common organisms were dermatophytes (61.66%), followed by NDMs (31.66%) and yeasts (6.66%). Greater prevalence of dermatophytes as the etiological agents was also seen in previous studies by Kaur et al. [15] in 2007, Aghamirian and Ghiasian [16] in Iran, and Sayed et al. [17] in Lebanon, while yeasts were the most common agents in a Canadian study in 2000 by Gupta et al. [18] and in a Greek study in 2002 by Koussidou et al. [19].

Amongst the NDMs,* A. niger *(9%) was the commonest isolate in our study, which was also found by Kaur et al. [15] and Grover [20]. In another study in Iran by Mikaeili and Karimi [7] in 2013,* A. flavus*,* A. niger*, and* A. fumigatus *were the more frequent isolated species.

*T*.* rubrum *was the most common dermatophyte isolated in our study followed by* T. verrucosum* and* T. mentagrophytes*. Kaur et al. [15] in their study in 2007 previously in the same institute had reported* T*.* mentagrophytes *as the most common dermatophyte, reflecting a changing trend in our area. Singal et al. [21] in their study in 2001 had also reported a change in the spectrum of dermatophytes in North India with most common isolate being* T. violaceum *(38%) followed by* M. audouinii*,* T. schoenleinii*,* T. tonsurans*,* M. gypseum*,* T. verrucosum*, and* T. mentagrophytes*. Our finding was in concordance with other studies by Lone et al. [11] in Kashmir, Alvarez et al. [22] in 2004 in Colombia, and Veer et al. [9] in 2007 in Aurangabad (India) which found* T*.* rubrum *as the most common dermatophyte, suggesting that* T. rubrum* might have developed increased virulence and better adaptation to hard keratin of skin, hair, and nail leading to its increased prevalence.

We have isolated many NDMs other than* Aspergillus *spp. (Table 4). In their study, Veer et al. [9] have isolated* Scopulariopsis *spp. (16.66%),* Alternaria *spp. (16.66%),* Fusarium *spp., and* Curvularia *spp. (8.3%) each. Sarma et al. [23] in New Delhi in 2008 had isolated* Curvularia lunata*,* Penicillium *spp.,* Alternaria alternata*, and* Geotrichum candidum* (1 each, 10%). Our study, to our knowledge, is probably the first to report many other NDMs like* Syncephalastrum *spp.,* Cunninghamella *spp.,* Rhodotorula *spp., and* A. terreus*, causing dermatomycoses. We have also isolated* Scytalidium *spp. and* Scopulariopsis *spp. and* Microsporum audouinii. Rhodotorula *spp. most commonly cause pulmonary and urinary tract infections, keratitis, and meningitis.* Syncephalastrum *spp. and* Cunninghamella *spp. can cause dermatomycoses and onychomycoses.* Scopulariopsis *spp. cause onychomycoses and rarely cause pneumonia and septicaemia.* A. terreus* causes disseminated and invasive aspergillosis mostly in osteomyelitis, meningitis, and endocarditis.* Scytalidium *spp. cause onychomycoses and other cutaneous and subcutaneous infections.

To our knowledge, ours is the first study reporting the mycological pattern incriminated in all the three hair, nail, and skin infections along with the isolation of various NDMs. However, there were limitations to our study: it is a retrospective analysis of data, many of the clinical details were not available to include in the study, and there was an inability of follow-up of patients in the final analysis of the precautions and medications, as it was difficult to trace the OPD patients.

## 5. Conclusion

The combined sensitivity of direct microscopy and culture, found to be better than direct microscopy and culture alone, reemphasizes the need for use of both tests as a routine for diagnosis and treatment of these patients. Furthermore, the demonstration of dermatophytes, NDMs, and yeasts in causing hair, nail, and skin infections in our study has reemphasized the increasing role of NDMs, thought to be normal laboratory or environmental contaminants, as a causative agent of dermatomycoses, replacing the lone causative dermatophytes. Increasing clinical acumen and suspicion and awareness of the demographic profile of the population involved along with more studies for better building of databases and knowing the trends of the etiological spectrum will go a long way in combating the disease occurrence and resulting cosmetic defacement.

## Figures and Tables

**Table 1:** Age and sex distribution of the patients.

Age in years	Male *n* (%)	Female *n* (%)	Total *n* (%)
0–10	21 (8.8%)	27 (23.4%)	48 (13.6%)
11–20	26 (11%)	13 (11.3%)	39 (11.1%)
21–30	56 (23.7%)	26 (22.6%)	82 (23.3%)
31–40	52 (22%)	20 (17.3%)	72 (20.5%)
41–50	33 (13.9%)	19 (16.5%)	52 (14.8%)
51–60	29 (12.2%)	9 (7.8%)	38 (10.8%)
>60	19 (8%)	1 (0.8%)	20 (5.6%)
Total	**236 (67.2%)**	**115 (32.7%)**	** 351 (100%)**

**Table 2:** Total samples received in the laboratory and correlation between sample type, KOH, and culture.

Samples	Total KOH+	KOH+, CUL−	CUL+, KOH−	Total CUL+	Both+	Both−
Skin (*n* = 123)	44 (35.7%)	9 (7.3%)	17 (13.8%)	48 (39%)	32 (26%)	62 (50.4%)
Hair (*n* = 32)	8 (25%)	2 (6.2%)	3 (9.3%)	9 (28.1%)	6 (18.7%)	21 (65.6%)
Nail (*n* = 196)	136 (69.3%)	4 (2%)	22 (11.2%)	144 (73.4%)	109 (55.6%)	41 (20.9%)
Total	**188 (53.5%)**	**15 (4.2%)**	**42 (11.5%)**	**215 (61.2%)**	**161 (45.8%)**	**110 (31.3%)**

**Table 3:** Total number of cases and culture positivity.

Type of sample	Total cases	Number of pathogen positivities *N* (%)		Mixed growth *N* (%)	No growth *N* (%)
	(*n* = 351)	Single growth	Double growth		
Skin	123 (35%)	48 (39%)	4 (3.2%)	4 (3.2%)	71 (55.9%)
Hair	32 (9.1%)	9 (28.1%)	0 (0%)	0 (0%)	23 (71.8%)
Nail	196 (55.8%)	144 (73.4%)	11 (5.6%)	5 (2.5%)	49 (23.4%)
		**205 (61.2%)**	**15 (4.2%)**	**9 (2.5%)**	**129 (36.7%)**
Total	**351**	**368**			

**Table 4:** Distribution of different isolates from different clinical samples.

	Organisms	Skin	Hair	Nail	Total *n* (%)
Moulds (36.1%)	*A. niger*	4 (3.1%)	—	29 (14%)	33 (9%)
	*A. terreus*	1 (0.7%)	—	1 (0.4%)	2 (0.5%)
	*A. flavus*	4 (3.1%)	—	26 (12.5%)	30 (8.1%)
	*A. fumigatus*	2 (1.5%)	—	8 (3.8%)	10 (2.7%)
	*Penicillium *spp.	2 (1.5%)	—	12 (5.7%)	14 (3.5%)
	*Fusarium *spp.	—	—	7 (3.3%)	7 (1.9%)
	*Mucor *spp.	2 (1.5%)	—	10 (4.8%)	12 (3.2%)
	*Syncephalastrum *spp.	1 (0.7%)	—	—	1 (0.2%)
	*Cunninghamella *spp.	—	—	1 (0.4%)	1 (0.2%)
	*Rhizopus *spp.	1 (0.7%)	—	9 (4.3%)	10 (2.7%)
	*Scopulariopsis *spp.	—	—	1 (0.4%)	1 (0.2%)
	*Scytalidium *spp.	—	—	1 (0.4%)	1 (0.2%)
	*A. alternata*	1 (0.7%)	1 (3.1%)	7 (3.3%)	9 (2.4%)
	*Bipolaris *spp.	—	—	1 (0.4%)	1 (0.2%)
	*Curvularia *spp.	—	—	1 (0.4%)	1 (0.2%)

Yeasts (8.6%)	*C. albicans*	7 (5.5%)	—	1 (0.4%)	8 (2.1%)
	*NAC*	7 (5.5%)	1 (3.1%)	15 (7.2%)	23 (6%)
	*Rhodotorula *spp.	—	—	1 (0.3%)	1 (0.2%)

Dermatophytes (13.8%)	*T. mentagrophytes*	2 (1.5%)	2 (6.2%)	4 (1.9%)	8 (2.1%)
	*T. rubrum*	8 (6.2%)	2 (6.2%)	7 (3.3%)	17 (4.6%)
	*T. schoenleinii*	5 (3.9%)	—	3 (1.4%)	8 (2.1%)
	*T. verrucosum*	4 (3.1%)	2 (6.2%)	6 (2.8%)	12 (3.2%)
	*T. violaceum*	1 (0.7%)	1 (3.1%)	2 (0.9%)	4 (1%)
	*Microsporum audouinii*	—	—	2 (0.9%)	2 (0.5%)

	Negative	71 (55.9%)	23 (71.8%)	49 (23.4%)	143 (38.8%)

	Contaminant	4 (3.1%)	—	5 (2.3%)	9 (2.4%)

	Total	**127**	**32**	**209**	**368**

## References

[1] Lacaz C. S., Porto E., Martins J. E. C., Heins-Vaccari E. M., Mello N. T., Lacaz C. S., Porto E., Martins J. E. C., Heins-Vaccari E. M., Mello N. T. (2002). Criptococcose. *Tratado de Micologia Médica*.

[2] De Araújo S. M., Fontes C. J. F., Leite Júnior D. P., Hahn R. C. (2012). Fungal agents in different anatomical sites in public health services in Cuiabá, state of Mato Grosso, Brazil. *Revista do Instituto de Medicina Tropical de São Paulo*.

[3] Agarwal U. S., Saran J., Agarwal P. (2014). Clinico-mycological study of dermatophytes in a tertiary care centre in northwest India. *Indian Journal of Dermatology, Venereology and Leprology*.

[4] Bhagra S., Ganju S. A., Kanga A., Sharma N. L., Guleria R. C. (2014). Mycological pattern of dermatophytosis in and around Shimla hills. *Indian Journal of Dermatology*.

[5] Adhikari L., Gupta A. D., Pal R., Singh T. S. K. (2009). Clinico-etiologic correlates of onychomycosis in Sikkim. *Indian Journal of Pathology and Microbiology*.

[6] Sujatha V., Grover S., Dash K., Singh G. (2000). A clinico-mycological evaluation of onychomycosis. *Indian Journal of Dermatology, Venereology and Leprology*.

[7] Mikaeili A., Karimi I. (2013). The incidence of onychomycosis infection among patients referred to hospitals in Kermanshah province, Western Iran. *Iranian Journal of Public Health*.

[8] Lakshmanan A., Ganeshkumar P., Mohan S., Hemamalini M., Madhavan R. (2015). Epidemiological and clinical pattern of dermatomycoses in rural India. *Indian Journal of Medical Microbiology*.

[9] Veer P., Patwardhan N. S., Damle A. S. (2007). Study of onychomycosis: prevailing fungi and pattern of infection. *Indian Journal of Medical Microbiology*.

[10] Gupta B. K., Kumar S., Kumar R. A., Khurana S. (1993). Mycological aspects of dermatomycosis in Ludhiana. *Indian Journal of Pathology and Microbiology*.

[11] Lone R., Bashir D., Ahmad S., Syed A., Khurshid S. (2013). A Study on clinico-mycological profile, aetiological agents and diagnosis of onychomycosis at a government medical college hospital in Kashmir. *Journal of Clinical and Diagnostic Research*.

[12] Shenoy M. M., Teerthanath S., Karnaker V. K., Girisha B. S., Krishna Prasad M. S., Pinto J. (2008). Comparison of potassium hydroxide mount and mycological culture with histopathologic examination using periodic acid-schiff staining of the nail clippings in the diagnosis of onychomycosis. *Indian Journal of Dermatology, Venereology and Leprology*.

[13] Das N. K., Ghosh P., Das S., Bhattacharya S., Dutta R. N., Sengupta S. R. (2008). A study on the etiological agent and clinico-mycological correlation of fingernail onychomycosis in eastern India. *Indian Journal of Dermatology*.

[14] Patel P., Mulla S., Patel D., Shrimali G. (2010). A study of superficial mycosis in south Gujarat region. *National Journal of Community Medicine*.

[15] Kaur R., Kashyap B., Bhalla P. (2007). A five-year survey of onychomycosis in New Delhi, India: epidemiological and laboratory aspects. *Indian Journal of Dermatology*.

[16] Aghamirian M. R., Ghiasian S. A. (2010). Onychomycosis in Iran: epidemiology, causative agents and clinical features. *Japanese Journal of Medical Mycology*.

[17] El Sayed F., Ammoury A., Haybe R. F., Dhaybi R. (2006). Onychomycosis in Lebanon: a mycological survey of 772 patients. *Mycoses*.

[18] Gupta A. K., Jain H. C., Lynde C. W., MacDonald P., Cooper E. A., Summerbell R. C. (2000). Prevalence and epidemiology of onychomycosis in patients visiting physicians' offices: a multicenter Canadian survey of 15,000 patients. *Journal of the American Academy of Dermatology*.

[19] Koussidou T., Devliotou-Panagiotidou D., Karakatsanis G., Minas A., Mourellou O., Samara K. (2002). Onychomycosis in Northern Greece during 1994–1998. *Mycoses*.

[20] Grover S. (2003). Clinico-mycological evaluation of onychomycosis at Bangalore and Jorhat. *Indian Journal of Dermatology, Venereology and Leprology*.

[21] Singal A., Rawat S., Bhattacharya S. N., Mohanty S., Baruah M. C. (2001). Clinico-mycological profile of tinea capitis in North India and response to griseofulvin. *Journal of Dermatology*.

[22] Alvarez M. I., González L. Á., Castro L. Á. (2004). Onychomycosis in Cali, Colombia. *Mycopathologia*.

[23] Sarma S., Capoor M. R., Deb M., Ramesh V., Aggarwal P. (2008). Epidemiologic and clinicomycologic profile of onychomycosis from north India. *International Journal of Dermatology*.

